# The hidden universe of human milk microbiome: origin, composition, determinants, role, and future perspectives

**DOI:** 10.1007/s00431-022-04383-1

**Published:** 2022-02-05

**Authors:** Alessandra Consales, Jacopo Cerasani, Gabriele Sorrentino, Daniela Morniroli, Lorenzo Colombo, Fabio Mosca, Maria Lorella Giannì

**Affiliations:** 1 Department of Clinical Sciences and Community Health, University of Milan, Milan, Italy; 2 Fondazione IRCCS Ca’ Granda Ospedale Maggiore Policlinico, NICU, Milan, Italy

**Keywords:** Human milk microbiome, Virome, Mycobiome, Origin, Determinants, Evolution

## Abstract

Although traditionally considered sterile, human milk is currently recognized as an alive ecosystem that harbors not only bacteria, but also viruses, fungi and yeasts, and minor genera, collectively known as the human milk microbiome (HMM). The seeding of HMM is a complex phenomenon whose dynamics are still a matter of research. Many factors contribute to its determination, both maternal, neonatal, environmental, and related to human milk itself. The transmission of microorganisms to the infant through breastfeeding may impact its present and future health, mainly shaping the GI tract microbiome and immune system. The existence and persistence of HMM as a conserved feature among different species may also have an evolutionary meaning, which will become apparent only in evolutionary times.

*Conclusion*: The complexities of HMM warrant further research in order to deepen our knowledge on its origin, determinants, and impact on infants’ health. The practical and translational implications of research on HMM (e.g., reconstitution of donor human milk through inoculation of infant’s own mother milk, modulation of HMM through maternal dietary supplementation) should not be overlooked.

**Table:** 

**What is Known:** *• Human milk harbors a wide variety of microorganisms, ranging from bacteria to viruses, fungi and yeasts, and minor genera.* *• Human milk microbiome is shaped over time by many factors: maternal, neonatal, environmental, and related to human milk itself.* *• The transmission of microorganisms through breastfeeding may impact the infant’s present and future health.*	
**What is New:** *• We provide an overview on human milk microbiome, hopefully encouraging physicians to consider it among the other better-known breastfeeding benefits.* *• Further studies, with standardized and rigorous study designs to enhance accuracy and reproducibility of the results, are needed to deepen our knowledge of the human milk microbiota and its role in newborn and infant’s health.*

**What is Known:**

*• Human milk harbors a wide variety of microorganisms, ranging from bacteria to viruses, fungi and yeasts, and minor genera.*

*• Human milk microbiome is shaped over time by many factors: maternal, neonatal, environmental, and related to human milk itself.*

*• The transmission of microorganisms through breastfeeding may impact the infant’s present and future health.*

**What is New:**

*• We provide an overview on human milk microbiome, hopefully encouraging physicians to consider it among the other better-known breastfeeding benefits.*

*• Further studies, with standardized and rigorous study designs to enhance accuracy and reproducibility of the results, are needed to deepen our knowledge of the human milk microbiota and its role in newborn and infant’s health.*

## Introduction

Human milk (HM) was traditionally thought to be sterile. However, the presence of bacteria in HM was never fully excluded. The first studies conducted between the end of the 19th and the beginning of the 20th century [1–3] focused on the potentially harmful nature of the bacteriological content of HM, failing to consider it, as it now is, as a precious resource. Still, in the late 60s, the presence of bacteria in HM was considered a consequence of low levels of personal and environmental hygiene [4].

Later on, in 2003, interest in the microbiology of HM resurfaced with a new perspective. Based on the detection of allegedly endogenous lactic acid bacteria from HM of eight healthy mothers, it was suggested that HM could be considered a symbiotic food, harboring safe bacteria with a potential role in the prevention of neonatal infectious diseases [5].

Over time, the development of culture-independent techniques (e.g., quantitative polymerase chain reaction and next-generation sequencing—NGS), in addition to the already well established culture-dependent ones, has progressively allowed for the characterization of the composition, diversity, and variability of HM microflora in greater detail, albeit with some limitations [6].

Today, HM is considered “mother nature’s prototypical probiotic food” [7]. Growing research on this subject has led to a deeper understanding of the matter, discovering that HM is an alive universe populated by bacteria, viruses, fungi and yeasts that cooperate for the present and future health of the infant. This complex host-associated microbial community constitutes the HM microbiome (HMM).

The aim of this review is to provide an overview of what is currently known on HMM origin, composition, determinants, and role, eventually suggesting possible future directions for researchers who want to further explore this field.

## Origin of HMM

The seeding of HMM is a complex and dynamic process, still not completely understood to date. Multiple, non-mutually exclusive, sources of HMM have been suggested (Table 1). It is still up for debate whether the mammary gland hosts a resident microbiome (i.e., the mucosal interface model) or it is simply a bystander subjected to a constant influx of microbes from exogenous sources (i.e., the constant influx model). This latter model is supported by the current lack of evidence of bacterial adhesion to the mammary epithelium outside of a mastitis setting, and of bacterial reproduction within the mammary tissue. Conversely, the mucosal interface model is supported by evidence of a pre-lactation mammary gland microbiome [8]. However, the fact that nonlactating mammary gland microbiome differs from HMM does not allow to exclude the constant influx model [9].

**Table 1:** Overview of the main hypothesized sources of HMM

Source	Supporting evidence	Alleged mechanism
Infant oral cavity	Oral bacteria (e.g., *Streptococcus salivarius*, *Streptococcus mitis*, *Rothia mucilaginosa*, and *Gemella* spp.) in HM [10]	Retrograde flow of milk from infant oral cavity to mammary ducts
Maternal skin	Human skin commensals (e.g., *S. epidermidis*, *Corynebacterium* spp. and Malassezia) in HM [11]	Colonization of mammary gland by maternal skin microbiota through the nipple
Maternal GI tract	Strict GI anaerobes (e.g., *Bifidobacterium*, *Bacteroides*, *Clostridium* [12]), and *Saccharomyces* [13] in HM	Internalization by dendritic cells during late pregnancy and lactation of live bacteria from the maternal GI tract, which then reach the mammary gland through lymphatic circulation (entero-mammary pathway) [14]

*HM* human milk, *GI* gastro-intestinal

## Composition

Although historically the knowledge of HMM was only limited to bacterial species [15], recent evidence highlighted that HM contains a wide variety of microorganisms, including viruses, fungi and yeasts, and new genera (Table 2).

**Table 2:** Composition of HMM

Microorganisms	Load	Main constituents
Bacteria	10^6^ cells/ml [13]	Two different “cores” hypothesized: - *Staphylococcus*, *Streptococcus*, *Serratia*, *Pseudomonas*, *Corynebacterium*, *Ralstonia*, *Propionibacterium*, *Sphingomonas*, and uncultured members of *Bradyrhizobiaceae* [16]; - *Staphylococcus*, *Streptococcus*, *Bacteroides*, *Faecalibacterium*, *Ruminococcus*, *Lactobacillus*, and *Propionibacterium* [17]
Viruses	-	- **Phages:** *Myoviridae*, *Siphoviridae*, and *Podoviridae* [18]; - **Eukaryotic viruses:** *Herpesviridae*, *Poxviridae*, *Mimiviridae*, and *Iridoviridae* [18]
Fungi and yeasts	2.5 to 3.5 × 10^5^ cells/ml [19, 20]	*Malassezia*, *Davidiella*, *Sistotrema*, and *Penicillium* [20]
Other	-	- **Protozoa:** *Toxoplasma gondii* and *Giardia intestinalis* (found in healthy women, without clinical sign of parasitic infection) [17]; - **Archaea:** *Methanobrevibacter smithii* and *Methanobrevibacter oralis* [21]

### Bacteriome

The implementation of the new NGS techniques, such as metataxonomics (16SrRNA gene sequencing) and metagenomics (shot-gun sequencing), has allowed for the detection of several new bacterial species, including many anaerobes, adding up to a total of more than 1300 different species [12, 16, 17, 22–27].

However, when trying to determine what constitutes the HM bacteriome, inter-individual variability, and geographic location of the study, methods used for HM collection, storage, and analysis must be taken into consideration. Hence, the definition, and the existence itself, of a “core” HM bacteriome is still a matter of debate [28].

Using genomic analysis, different studies have detected a wide variety of soil and water-related microorganisms, such as *Bradyrhizobium*, *Pseudomonas*, and *Stenotrophomonas* [8, 12, 16, 22, 26, 29]. However, these results must be critically interpreted, as such microorganisms could also be contained in molecular biology reagents, solutions, and kits, and their relative amounts could be amplified by DNA techniques, thus contributing to mistaken interpretations [22, 30–32]. Furthermore, differentiating between live or dead microorganisms is critical. Therefore, appropriate techniques should be selected to limit possible biases [33].

### Virome

Most (95%, [18]) of the HM virome is made of bacteriophages, with eukaryotic viruses and other viral particles constituting a lesser proportion.

HM virome has distinctive features that differentiate it from other viromes (e.g., adult stool, urine, saliva, and cerebrospinal fluid viromes) [34, 35]. Conversely, a significant number of shared viruses have been identified between HM and infant stool from mother-infant pairs, supporting their vertical inheritance through breastfeeding [34, 36]. Interestingly, it has been noted [34] that the virome of infant stool bears a closer resemblance to HM than to adult stool.

### Mycobiome and other -omes

Fungi are an important component of the human microbiome [37]. However, their presence in HM is a relatively recent discovery [20]. Although considering geographical variability, the existence of a core mycobiome has been hypothesized, thus suggesting that their transmission through HM is a conserved feature.

Other microorganisms, until recently neglected, contribute to the HMM. In particular, current research has been focusing on Archaea. The presence of archaeal DNA has been demonstrated in 8/10 HM samples analyzed, none of which belonging to women with mastitis, thus suggesting a protective role [17]. Conversely, other authors did not identify archaeal DNA in the HM samples analyzed [38].

## Determinants Of HMM

**Fig. 1:**
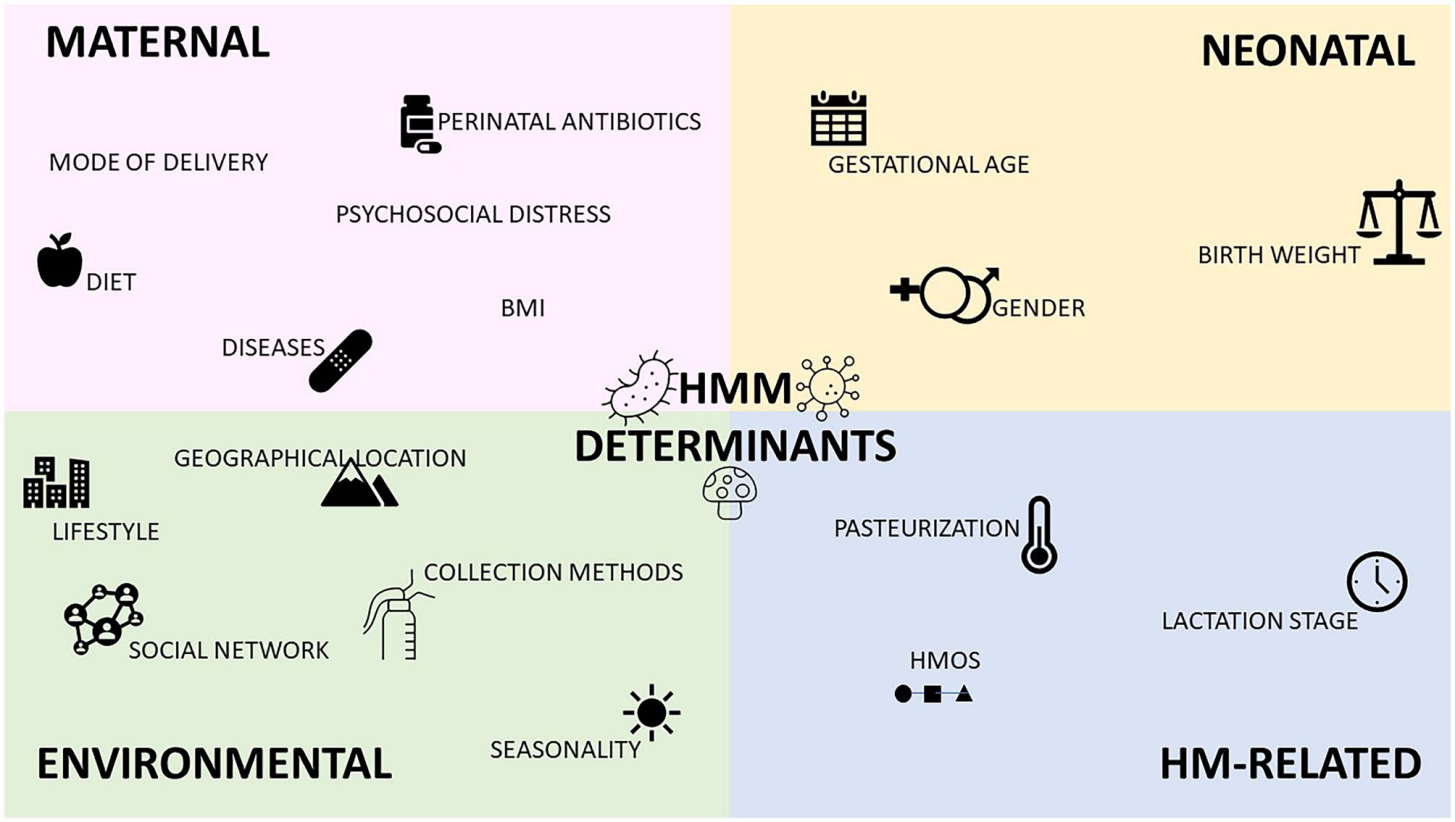
Overview of the main determinants of HMM (see text for explanation)

The complex HM ecosystem appears to be shaped over time by many factors: maternal, neonatal, environmental, and related to HM itself (Fig. 1). The extremely dynamic nature of HMM composition may account for the often-contradictory data reported in the Literature. Furthermore, it should be noted that many factors that have been implicated in the determination of HMM are closely intertwined.

## Maternal determinants

Some authors [26, 39–41] demonstrated that, compared to women who underwent a C-section, vaginally delivered women’s HM samples showed higher bacterial diversity and richness, with higher levels of *Bifidobacterium* and *Lactobacillus* spp. However, other studies did not confirm such results [42, 43]. A potential influence of the mode of delivery on the virome and mycobiome of HM has been hypothesized as well [44, 45].

A decrease in the abundance of *Lactobacillus*, *Bifidobacterium*, *Staphylococcus*, and *Eubacterium* spp. in HM samples from mothers who received peri-natal antibiotics has been reported [8, 46, 47]. Maternal chemotherapy during lactation has also been associated with a reduction in HM bacterial diversity [48].

Maternal diet impacts HMM composition (allegedly more so during pregnancy than during lactation [49–51]). High-fiber and high-fat food dietary regimens [49] as well as vitamin intake (vitamin C and complex B vitamins) [51] have been shown to alter HMM composition. Furthermore, both pre-pregnancy BMI and weight gain during gestation are reflected in differential abundances of bacterial strains (mainly *Streptococcus*, *Staphylococcus*, and *Bifidobacterium*) in HM [40, 52–54].

Compared to healthy women, mothers with celiac disease have lower levels of *Bacteroides* spp. and *Bifidobacterium* spp. in their milk [55]. Likewise, mastitis determines modifications in bacterial load and microbial diversity in HMM, which subside once clinical symptoms disappear [56–58].

Maternal post-natal psychosocial distress (defined as symptoms of anxiety, stress, or depression during the postnatal period) has been linked to lower HM bacterial diversity at 3 months post-delivery, with a progressive decrease in the relative abundance of *Staphylococci* and a parallel increase of some minority genera (*Lactobacillus*, *Acinetobacter*, and *Flavobacterium*) in mothers with low psychosocial distress [59].

## Neonatal determinants

Lower counts of *Enterococcus* spp. and higher counts of *Bifidobacterium* spp. have been detected in HM samples from mothers who delivered at term compared to preterm mothers [39]. Conversely, other authors [42] did not detect any difference in microbial profiles based on length of gestation, postulating a fail-safe mechanism that allows the mother to be “ready” to pass along her bacterial imprint regardless of gestational age at birth, as part of an evolutionary pressure directed towards the baby’s benefit. Variations in HM virome and mycobiome composition according to gestational age and birth weight have been recently demonstrated [44, 45].

Effect of newborn gender on HMM composition has been hypothesized [60], based on the detection of more *Streptococci* and less *Staphylococci* in HM from mothers of male infants compared to mothers of female infants. However, such differences have not been confirmed by other studies [42, 61].

## Environmental determinants

The analysis of HM samples collected from selected populations in Europe, Africa, and Asia, suggested that HMM composition is related to the geographical study location [62]. Furthermore, a high variability in HM metabolites across study sites, and an association between variations in HM metabolome and specific features of HMM, have been documented [63]. However, a novel analysis of HM samples from Ethiopia, The Gambia, Ghana, Kenya, the USA, Peru, Spain, and Sweden, demonstrated that, while HM bacterial communities varied geographically, they consistently contained the core genera *Staphylococcus* and *Streptococcus* [64]. Such results have been confirmed by a recent systematic review [65], which included twelve studies that used culture-independent methods to identify bacteria at genus level in HM from healthy women. Notably, it has been speculated that at least part of the geographical variability in HMM composition might be related to differences in the setting and procedure of HM collection, storage, and analysis [66]. As for collection methods, it has been observed [61] that HM from mothers who use breast pumps have higher microbial load and lower abundance of cultivable staphylococci compared to HM samples collected manually. Conversely, other authors found no difference in ɑ-diversity between samples collected by manual expression or by pumping with a single-use sterile device [67].

The analysis of HMM from women living in the same Indian region but with different lifestyles (traditional vs. western-like), revealed that HM samples from “rural women” had higher diversity and greater abundance of sub-dominant bacterial lineages than those from “urban women” [68].

A study conducted in the Central Africa Republic within a small-scale society suggested that seasonality may influence the relative abundance of specific taxa in HMM, although it may be difficult to determine whether the variation in composition depends on differences in seasonal environmental exposure and/or seasonal variation in diet [69]. The same study [69] explored the relationship between mother-infant social network size, and HMM composition and diversity, showing how HM from mothers with larger networks, and infants with more caregivers, had higher microbial evenness (but not microbial richness) than HM from mothers whose infants had fewer caregivers.

## HM determinants

Cabrera-Rubio et al. [26] were the first to describe the changes HMM undergoes over time, from colostrum to transitional and mature milk. These authors reported a progressively increased abundance of typical oral inhabitants (e.g., *Veillonella*, *Leptotrichia*, and *Prevotella* spp.) in transition and mature HM, and higher counts of *Bifidobacterium* at later stages of lactation. Other authors [39] later reported a greater influence of lactation stage on *Bifidobacterium* and *Enterococcus* spp. counts, which showed a progressive increase in their concentration from colostrum to mature HM, as did *Lactobacillus* and *Staphylococcus* spp. Different patterns over time have been described. Analyzing HM samples collected at 3 time points over a 4-week interval, a set of 9 “core operational taxonomic units” was identified [16]. However, in some samples, HM bacterial communities were rather consistent over time, whereas, in others, the relative abundance of the bacterial genera shifted over time [16]. Some authors [60] observed a relative stability of HMM over time, with only small changes in some minority genera, while others [43] did not observe any effect of lactation stage on HMM composition. Regarding the virome, it was recently documented [44] that, although bacteriophages are predominant in both transient and mature HM samples, transient HM has a greater abundance of *Podoviridae* and *Myoviridae*, whereas in mature HM *Podoviridae* decreases, and *Siphoviridae* becomes the most abundant family. As for mycobiome, a recent study [45] analyzed samples of HM from different stages of lactation and found that, in transient HM samples, *Saccharomyces cerevisiae* and *Aspergillus glaucus* were the most abundant species, while *Penicillium rubens* and *Aspergillus glaucus* were predominant in mature HM samples.

It has been speculated that other HM components, such as HM oligosaccharides (HMOs—prebiotics), milk fatty acids, hormones, immune cells, and antibodies, could modulate the composition of HMM [70, 71]. In particular, HMOs may promote the growth of Staphylococcus spp. in the lactating mammary gland [72].

## Donor human milk and HMM

When mother’s own milk is not available or insufficient, donor HM (DHM) is the second-best alternative [73–75]. However, pasteurization, needed to guarantee the necessary microbiological safety standards, inevitably inactivates several of HM nutritional and biological properties [76], including HMM. As a matter of fact, pasteurization eliminates most milk bacteria (except the spore-forming *Bacillus* species [77–79]). Nevertheless, viability of HMM is no longer considered essential. Indeed, the probiotic effect of beneficial microbes in HM has been hypothesized to rely on the ability of the host’s cells to recognize specific bacterial components or products, thus activating the immune system. These “non-viable (more often heat-inactivated) microbial cells (intact or broken) or crude cell extracts (i.e., nucleic acids, cell-wall components)” are known as para-probiotics or ghost probiotics [80].

## Role and benefits of HMM

HMM seeds the infant GI tract with pioneering bacteria, thus contributing to the establishment of both the infant oral and gut microbiota [81, 82]. However, not all the bacteria present in HM are found in the infant gut, but, rather, only a select few seem to colonize the newborn [42]. Nonetheless, it has been hypothesized that transient exposure could be just as effective as persistent colonization [83, 84]. Moreover, bacteria in HM may upregulate protective factors such as antibodies, immune cells, lactoferrin, and beta-defensins that would then be passed on to the neonate through breastfeeding [42]. The HM virome, especially bacteriophages, likely contributes to the gut ecology of the infant, as well [18].

Early microbial exposure is essential to provide antigenic stimuli that promote the intestinal immune system maturation by encouraging a shift from the predominant intrauterine T helper (TH) 2 cell immune milieu to a TH1/TH2 balanced response, and triggering regulatory T cell differentiation [85].

Through modifications of the infant gut microbiota and by means of the gut-brain axis, HMM may also influence the development of a more convenient behavioral phenotype of the offspring, as hypothesized for other HM bioactives [86]. Indeed, in early infancy, HM may promote the colonization of a specific microbiota that influences offspring biobehavioral regulation. A milk-oriented infant gut microbiota may produce a less energetically costly behavioral phenotype in order to more optimally allocate maternal energetic investment [86].

An association between breastfeeding and upper respiratory microbiota composition at 6 weeks was reported, with breastfed infants showing a significantly different microbial composition than formula-fed ones [87]. Interestingly, such association seems to disappear at 6 months of age (when weaning typically begins) [87, 88].

Finally, it has been hypothesized that HMM may benefit the mother too, protecting her against infections such as mastitis [42].

## Potential evolutionary significance of HMM

Breastfeeding represents a valuable route of maternal microbial transmission both in humans and other animals (i.e., rhesus monkeys, cows, sheep, goats) [89–92]. Since the transmission of HMM appears to be a conserved feature among different species, a possible evolutionary purpose can be hypothesized.

Maternal microbial transmission provides offspring with important microbes early in life, rather than leaving their acquisition to chance during later stages of development. By shaping the offspring’s own microbiome, such microbes may determine evolutionary advantages in the recipient [11, 93, 94]. Consequently, within a broader evolutionary context, HMM transmission could be seen as at least partially capable of shaping the microbiome of the whole species over evolutionary time, since microbes that promote host fitness will increase their odds of reaching the next generation.

## Future directions

Despite the progress made in the last decades, many unanswered questions still remain. However, the lack of internationally recognized “best practices” in HMM analysis (e.g., HM collection, storage, processing, DNA extraction, and sequencing) often limits comparison among studies. Therefore, standardized and rigorous study designs are needed to promote accuracy and reproducibility of the results.

Many topics addressed in the present review represent interesting fields to explore. Firstly, the sources and pathways of HMM seeding should be further examined, possibly through experimental studies on animal models. Moreover, interactions between mother, infant, and environment should be better investigated, thus uncovering hidden mechanisms of coregulation between different microbiomes. Additionally, all the members of the microbial community of HM should be equally considered. So far, bacteria have been the most studied microorganisms. Progressively, attention has shifted to viruses (although with a strong bias towards DNA viruses), fungi, and yeasts. The next frontier will be to explore the archaeome and to deepen our knowledge of the potential infant health implications of the “minor” components of HMM. Finally, the functional significance of HMM and its impact on infants’ GI tract microbiome, immune system, and later health would benefit from appropriate experimental, possibly longitudinal, studies.

The practical and translational implications of research on HMM should also be considered. For example, studies on the reconstitution of DHM through inoculation of definite amounts of infant’s own mother milk aimed at restoring the live HMM, as described by Cacho et al. [95], should be incentivized. Likewise, the possible role of maternal dietary supplementation with pre- or postbiotics aimed at modulating HMM should be clarified, as well as the more suitable timing for such supplementation (e.g., during pregnancy and/or during lactation).

## Conclusions

Although traditionally considered sterile, it is now clear that HM harbors a wide variety of microorganisms, ranging from bacteria to viruses, fungi and yeasts, and minor genera. The transmission of such microorganisms to the infant may help determine its present and future health, mainly shaping the neonatal GI tract microbiome and immune system. The complexities of the HM ecosystem warrant further research to deepen our knowledge on HMM origin, determinants, and implications for infants’ health.

## Abbreviations

BMI: Body mass index
DHM: Donor human milk
GI: Gastro-intestinal
HM: Human milk
HMM: Human milk microbiome
HMOs: Human milk oligosaccharides
NGS: Next-generation sequencing
